# An Integrated Approach to the Taxonomic Identification of Prehistoric Shell Ornaments

**DOI:** 10.1371/journal.pone.0099839

**Published:** 2014-06-17

**Authors:** Beatrice Demarchi, Sonia O'Connor, Andre de Lima Ponzoni, Raquel de Almeida Rocha Ponzoni, Alison Sheridan, Kirsty Penkman, Y. Hancock, Julie Wilson

**Affiliations:** 1 BioArCh, Department of Archaeology, University of York, York, United Kingdom; 2 School of Life Sciences, University of Bradford, Bradford, United Kingdom; 3 Department of Physics, University of York, York, United Kingdom; 4 National Museums Scotland, Chambers Street, Edinburgh, United Kingdom; 5 BioArCh, Department of Chemistry, University of York, York, United Kingdom; 6 Departments of Mathematics and Chemistry, University of York, York, United Kingdom; 7 York Centre for Complex Systems Analysis (YCCSA), University of York, York, United Kingdom; Natural History Museum of Denmark, Denmark

## Abstract

Shell beads appear to have been one of the earliest examples of personal adornments. Marine shells identified far from the shore evidence long-distance transport and imply networks of exchange and negotiation. However, worked beads lose taxonomic clues to identification, and this may be compounded by taphonomic alteration. Consequently, the significance of this key early artefact may be underestimated. We report the use of bulk amino acid composition of the stable intra-crystalline proteins preserved in shell biominerals and the application of pattern recognition methods to a large dataset (777 samples) to demonstrate that taxonomic identification can be achieved at genus level. Amino acid analyses are fast (<2 hours per sample) and micro-destructive (sample size <2 mg). Their integration with non-destructive techniques provides a valuable and affordable tool, which can be used by archaeologists and museum curators to gain insight into early exploitation of natural resources by humans. Here we combine amino acid analyses, macro- and microstructural observations (by light microscopy and scanning electron microscopy) and Raman spectroscopy to try to identify the raw material used for beads discovered at the Early Bronze Age site of Great Cornard (UK). Our results show that at least two shell taxa were used and we hypothesise that these were sourced locally.

## Introduction

Mollusc shells appear to have been among the first durable materials used for personal ornaments and building tools [1]–[5]. Shells and shell ornaments found in archaeological sites [6]–[11] have helped shape our understanding of the interactions between past peoples and their environment [12]–[16]. In the Upper Palaeolithic, taxonomically identifiable perforated shell assemblages appear to have been selected on the basis of durability, size and shape as well as rarity or colour, but with large freedom in the choice of taxa used [17]. The thorny oyster *Spondylus* has special symbolic and cultural significance for the Holocene peoples of both the Old and New World [18]–[21].

Unfortunately, worked or degraded artefacts are difficult to identify; to date the only approach to aid identification of such shell fragments has been microstructural analysis [22]–[23]. Shells preserve organic molecules trapped within the mineral skeleton, particularly proteins that are responsible for the process of biomineralisation [24]–[28]. These proteins have been exhaustively studied in amino acid geochronology (Amino Acid Racemisation dating, AAR) [29]–[35]. Differences in bulk amino acid composition between taxa of mollusc shells have been observed to result in different rates of protein degradation (racemisation) [36]–[39]. Further, these composition differences have been used as a taxonomic identification tool for mollusc shells [40]–[41] and foraminifera [42].

Here we present a refined version of this idea for the identification of molluscan taxa, based upon the bulk amino acid composition of the intra-crystalline protein (IcP) fraction. The amino acid composition of the intra-crystalline organic matrix is different from the inter-crystalline matrix (between crystallites). Isolation of the IcP fraction by strong oxidation [43]–[46] ensures that taphonomically induced compositional variation is minimised. Here we investigate the relationship between the IcP bulk amino acid composition of 29 different molluscan taxa using statistical classification techniques.

We sampled six of the tiny disc shell beads from the Early Bronze Age necklace (or chest ornament) found at Great Cornard, Suffolk [47] and applied our approach to the identification of the raw material used for their manufacture. We integrated the biomolecular approach with macro- and micro-morphological observations (by light microscopy and scanning electron microscopy) and mineralogical information obtained by Raman spectroscopy.

## Materials and Methods

### 2.1 Amino acid analysis

The North East Amino Acid Racemisation (NEaar) laboratory is a geochronological facility dedicated to the analysis of chiral amino acids from biominerals, including mollusc shells, for dating purposes. In this study we exploit the IcP bulk amino acid compositional data from the NEaar database. The dataset used here comprised 777 samples, each analysed in duplicate by reverse-phase high-pressure liquid chromatography (RP-HPLC). Table 1 gives the details of the molluscan taxa considered and their taxonomic classification; we used the taxonomy reported in the online World Register of Marine Species [48] and in the database AnimalBase [49]. Due to the large number of undetermined species in the dataset, we refer to samples by genus and not species, and only attempt to classify to this taxonomic level. Samples are from a range of geographical locations and ages from modern to ∼2 Ma. As both temperature and time affect the extent of protein degradation, we consider their possible effects on the compositional signal in section 3.2. All shells and the shell beads from the site of Great Cornard were prepared and analysed using the protocol detailed below.

**Table 1:** Details of the molluscan taxa.

Class	Order	Family	Genus
Bivalvia (155)	Arcoida (17)	Glycymerididae (17)	*Glycymeris* da Costa, 1778 (17)
	Ostreoida (10)	Ostreidae (6)	*Ostrea* Linnaeus, 1758 (6)
		Spondylidae (4)	*Spondylus* Linnaeus, 1758 (4)
	Pectinoida (25)	Pectinidae (25)	*Pecten* O.F. Müller, 1776 (25)
	Unionoida (18)	Margaritiferidae (12)	*Margaritifera* Schumacher, 1815 (12)
		Unionidae (6)	*Unio* Philippson, 1788 (6)
	Veneroida (73)	Arcticidae (19)	*Arctica* Schumacher, 1817 (19)
		Cardiidae (17)	*Cardium* Linnaeus, 1758 (17)
		Cyrenidae (21)	*Corbicula* Magerle von Mühlfeld, 1811 (21)
		Tellinidae (8)	*Macoma* Leach, 1819 (8)
		Veneridae (8)	*Dosinia* Scopoli, 1777 (3)
		Veneridae (8)	*Mercenaria* Schumacher, 1817 (5)
	Mytiloida (12)	Mytilidae (12)	*Modiolus* Lamarck, 1799 (12)
Gastropoda (620)	Littorinimorpha (244)	Bithyniidae (104)	*Bithynia* Leach, 1818 (104)
		Littorinidae (54)	*Littorina* Férussac, 1822 (54)
		Rissoidae (2)	*Rissoa* Desmarest, 1814 (2)
		Strombidae (84)	*Conomurex* Bayle in P. Fisher, 1884 (84)
	Hygrophyla (15)	Lymnaeidae (8)	*Lymnaea* Lamarck, 1799 (8)
		Planorbidae (7)	*Planorbarius* Duméril, 1805 (5)
			*Anisus* Studer, 1820 (2)
	Neogastropoda (9)	Muricidae (9)	*Nucella* Röding, 1798 (9)
	Archaeogastropoda (172)	Patellidae (172)	*Patella* Linnaeus, 1758 (172)
	Stylommatophora (48)	Helicidae (9)	*Cepaea* Held, 1837 (9)
		Pupillidae (24)	*Pupilla* J. Fleming, 1828 (24)
		Hygromiidae (15)	*Trochulus* Chemnitz, 1786 (15)
	Caenogastropoda (20)	Cyclophoridae *incerta saedis* (20)	*Cyclophorus* Montfort, 1810 (20)
	Subclass:Vetigastropoda (19)	Trochidae (19)	*Phorcus* Risso, 1826 (19)
	Infraclass: [unassigned] Heterobranchia (93)	Valvatidae (93)	*Valvata* (93)
Scaphopoda (2)	Dentaliida (2)	Dentaliidae (2)	*Antalis* H. Adams & A. Adams, 1854 (2)

The number of biological replicates available for each taxonomic level (genus, family, order if available, and class) is given in parentheses.

For the shell dataset, no specific permissions were required for these locations. The field studies did not involve endangered or protected species. All collaborators have agreed to the use of data from their samples for the purpose of this study. For published data, details of each study location are available in the publications listed in SI-5 (samples are identifiable through their unique identifier, the NEaar number). For unpublished data, details are available upon request. No permits were required for the analyses on the beads from the excavation at Great Cornard (TL 8580 9670), conducted by Suffolk Archaeology.

Intra-crystalline amino acid signatures were obtained by preparing samples for the analysis of total hydrolysable amino acids (THAA) according to the method detailed in Penkman et al. [44]. Briefly, this involves: powdering a sub-sample taken from a shell specimen (∼2–3 mg) with pestle and mortar; soaking the powders in sodium hypochlorite (12% w/v) for 48 h; rinsing the bleach off with ultrapure water; hydrolysing the peptide bonds by exposing the dried powders to harsh acidic conditions (7 M HCl, 24 hours, at 110°C); evaporating the samples to dryness and finally rehydrating them with a solution containing an internal standard (the non-protein amino acid L-homo-arginine) for quantification. Rehydrated samples are analysed in duplicate by RP-HPLC, using a modified method of Kaufman and Manley [50] that allows the routine analysis of L- and D- enantiomers. Here we consider the amino acids that are eluted with optimal chromatographic resolution: Asx (aspartic acid/asparagine), Glx (glutamic acid/glutamine), Ser (serine), Gly (glycine), Ala (alanine) and Val (valine).

### 2.2 Statistical Methods

Principal Components Analysis (PCA) was used for data visualisation. The original axes corresponding to the six variables (the concentrations of the six amino acids) are rotated to give new variables, or principal components, such that the first principal component lies in the direction of the maximum variance in the data. This provides a one-dimensional approximation to the data that retains the maximum information possible. Better approximations are obtained by using further principal components, where the *k*th principal component is orthogonal to each of the first (*k −*1) components and captures the maximum variance not already accounted for by these components. As most of the information in the data is captured in the first few principal components, scores plots showing the new coordinates in just two or three dimensions can be used to show the distribution of the data.

We used Learning Vector Quantization (LVQ) for classification [51]. An LVQ neural network divides the input space into areas (Voronoi cells) each associated with a particular class in the training data (although multiple cells may have the same class). During training the cells are adjusted to give the best classification boundaries with the aim of concentrating the information in the training data into a reasonably small set of prototype vectors representing each class. Comparison with these prototype vectors allows new samples to be classified.

Kaufman et al. [41] used the coefficient of similarity (CS), defined by 
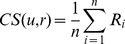
to compare the data from an unknown shell, *u*, with a reference shell, *r*. Here *n* is the number of variables (6 in our study) and 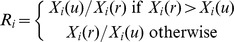
with *X_i_* denoting the *i*th variable. The CS tends to unity as the similarity between samples increases and the reference sample that provides the best match to the unknown sample is considered a possible classification.

### 2.3. Optical microscopy

Low magnification, reflected light microscopy was undertaken using a Wild Heerbrugg M8 stereomicroscope (6× to 50×) and a Dino-Lite Premier HR, 5 Mp digital microscope with polarizer (AM-7013MZT) (25× to 50×, 200× to 250×).

### 2.4. Scanning Electron Microscopy

An FEI Quanta 400 with eSEM capability and a Low Vacuum mode was used for the SEM imaging. The fracture surfaces of the beads and comparative shell specimens (attached to 12 mm aluminium stubs by double-sided tape) were imaged under low vacuum with a spot size of up to 6.5 units and an accelerating voltage of 20 kV. The cut sections of some comparative shell specimens were mounted on 12 mm aluminium stub using a glue gun. These were ground, polished, etched with dilute acetic acid, rinsed in deionised water, dried and gold plated to ∼16 nm using the Emitech K550 sputter coater, then imaged under high vacuum with a spot size 3 and an accelerating voltage of 20 kV.

### 2.5. Raman spectroscopy

A HORIBA XploRA instrument with 532 nm laser wavelength and ×100/0.75 NA objective in confocal mode was used for Raman spectroscopy. Spectra were obtained using the HORIBA LabSpec software set at 1 s laser exposure and resulting in ∼3.5 mW power at the sample with each measurement averaged over 40 spectral acquisitions.

## Results

### 3.1 Amino acid data normalisation

In order to compare amino acid concentrations between different samples, some form of normalisation must first be performed. Absolute values (scaled according to an internal standard) require very accurate measurement, while relative concentrations (expressed in terms of the total concentration) suffer from interdependency; measurement error on any one amino acid will affect the other concentrations. Previous studies have used ratios to describe the amino acid composition of molluscan fossils, due to the difficulties in comparing either absolute or relative concentrations [40]–[41]. However, the use of ratios (expressed as fractions) suffers from the fact that small and possibly unreliable values become very important when appearing in the denominator and can then dominate the analysis. We found that use of relative concentrations gave the best classification results (on independent test data), but rather than evaluating each as a percentage of the total amino acid concentration, we normalised so that the sum of the six amino acid concentrations was the same value for each sample. The resulting compositions were used as variables in subsequent analysis, with each sample represented by a feature vector of length six.

### 3.2. The effect of age and geographical region on amino acid concentration

As a fossil dating technique, AAR utilises the fact that the D/L value of amino acids increases with age, i.e. the [D] concentration increases and the [L] concentration decreases until D/L  = 1. This has been applied successfully to date a range of depositional environments, from fluvial terraces to coastal raised beaches and shell middens [30]–[33], [52]–[53]. As we are considering a closed system of proteins (the IcP fraction), loss (by leaching or diffusion) from this system should be minimal (<5%). This has been verified in a range of molluscan genera [44]–[45]. However, the compositional signal of fossil shells may still be affected by diagenesis, particularly amino acid decomposition (for example, serine dehydration to alanine [54]), and this may confound any taxonomic signal. To investigate this, we consider the genera for which we have examples of different ages. The normalised amino acid composition data of 78 *Valvata piscinalis* samples from the UK, with ages ranging from 500 to 600,000 years, were analysed, but only Glx appeared to show a consistent trend with age. Similarly, the variability seen for three other genera (*Arctica*, *Littorina* and *Margaritifera*) for which data was available for multiple age groups, does not allow such effects to be modelled (Supporting Information S1).

**Figure 1:**
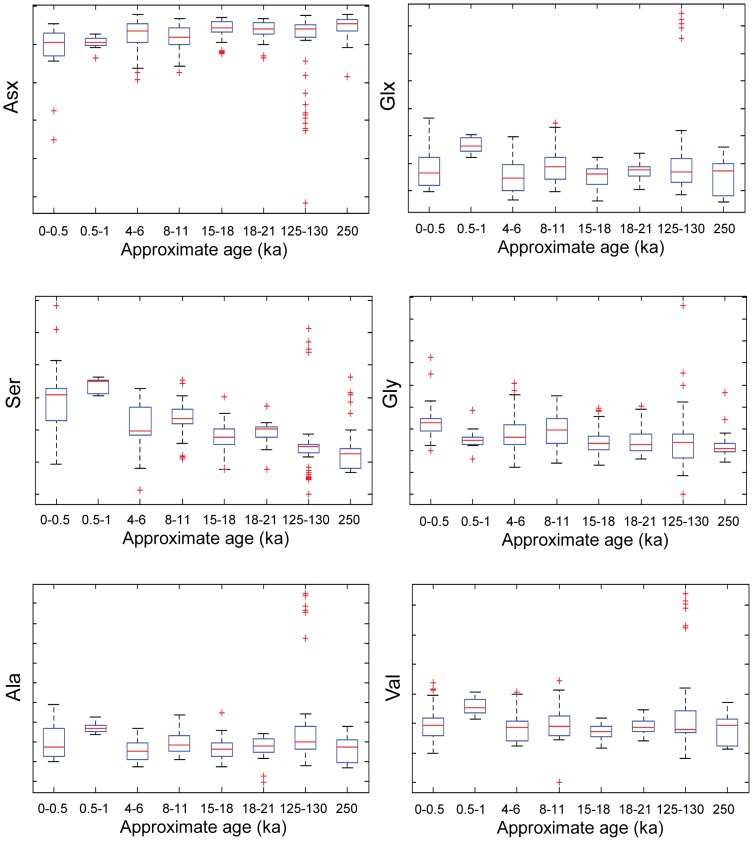
Amino acids distributions for *Patella*. Boxplots showing the distribution of concentrations with age for individual amino acids in shells of genus *Patella*. Concentrations are relative (y-axis units are arbitrary), having been normalised so that the sum over the six amino acids is the same for each sample. For each age group, the rectangular box shows the inter-quartile range with the median indicated by the line inside. The "whiskers" extending from each box show the maximum/minimum values unless these extend more than 1.5 times the inter-quartile range: any examples beyond this are indicated by crosses.

**Figure 2:**
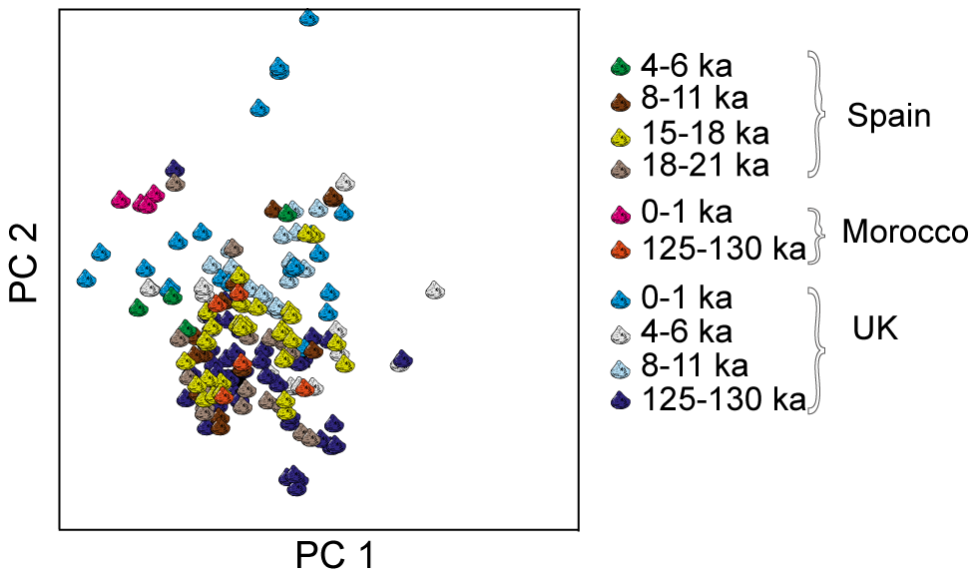
PCA scores plot for *Patella*. Scores plot for the first two principal components obtained from data for shells of the *Patella* genus. The plot shows no consistent pattern with either age or country of origin. Age bins are given in thousand years.

Temperature can also affect the extent of diagenesis, and differences due to age could be confounded by differences in the geographical region of origin. The *Patella* data, obtained from shells collected in the UK, Spain and Morocco, were used to investigate the relationship between location and amino acid composition. For most amino acids in *Patella*, the overall distribution of concentrations with age remain stable over time, with only Ser showing a pronounced trend with age (Figure 1). The PCA scores plot for the first two principal components (together accounting for over 95% of the total variance in the data) shows no clustering associated with either age or geographical region (Figure 2). The shells of the *Patella* genus used for this analysis had either been identified as *Patella vulgata* or were of undetermined species. Differences at species level could potentially obscure any association with age or thermal history (geographic location).

We conclude that although temperature and age are likely to affect the amino acid composition, this cannot be modelled effectively.

### 3.3 Classification based on amino acid concentration

As no consistent pattern could be found with either geographical location or age, we made no attempt to model the effect of such diagenetic changes on amino acid concentration. The six normalised variables were used for classification by Learning Vector Quantization (LVQ) and for Coefficient of Similarity (CS) calculations.

As with the discriminant analysis used by Andrews et al. [40], both the LVQ algorithm and the CS method of Kaufman et al. [41] require data for training and, as supervised methods, need to be validated using test data, not used for training, to prevent over-fitting. When few examples are available, as is the case for some genera here, the use of a separate test set can be a problem; the more examples used for training, the better the classification is likely to be, but error estimate from a small test set is likely to be unreliable, with a lucky choice of test data resulting in an over-optimistic estimate and an unlucky choice being too pessimistic. To overcome this problem and allow training with as many examples as possible, we used leave-one-out cross validation. This approach uses one example for validation and the rest of the data for training. The process is repeated, leaving out a different example each time, until every example has been used for validation.

**Figure 3:**
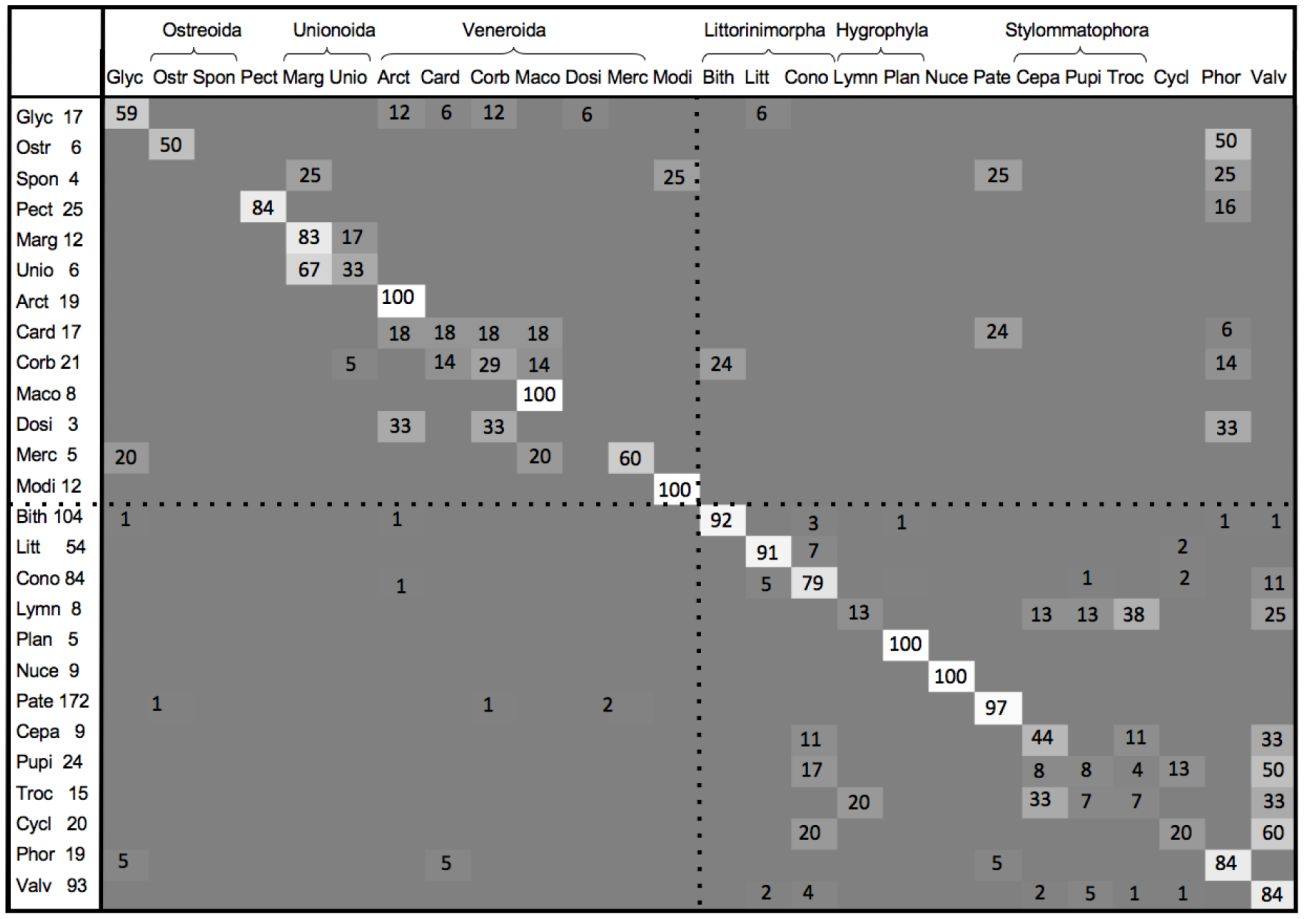
Classification results. Results of the classification performed using Learning Vector Quantization. Leave-one-out (L-O-O) classification was used for validation and the results show how the test samples were classified. Each row represents one of the 26 genera included in the analysis, as indicated on the left of the table together with the number of examples tested. The four-letter codes uniquely identify each genus (full names in Table 1). The columns show the predicted genera. Numerical values are percentages so that the element in column *i* of row *j* shows the percentage of genus *j* that were classified as genus *i* and the main diagonal shows the percentage of each genus correctly classified. The dotted lines separate Bivalvia from Gastropoda and brackets above the predicted class names group genera within the same order. The grey-scale intensities are related to the numerical values with white corresponding to 100% and the darkest grey to 0% (for which no numerical value is given). Note that rounding may result in rows not summing to 100%.

To assess the classification based on amino acid concentration, we used the data for 26 genera in training and validation. With just two examples each, *Anisus*, *Rissoa* and *Antalis* were not included in this analysis. The results of the LVQ classification are shown in Figure 3. Each row of the table shows the validation results for a particular genus. The columns show the predicted genera for these examples, so that the element in column *i* of row *j* shows the percentage of genus *j* that were assigned to genus *i* and the main diagonal shows the percentage of each genus correctly classified. Where no numerical value is given, no examples were assigned. We have used grey-scale intensities to emphasize areas of the table where genera are confused in the classification.

Figure 3 shows that some genera classify well (*Pecten*, *Margaritifera*, *Arctica*, *Macoma*, *Modiolus*, *Bithynia*, *Littorina*, *Planorbarius*, *Nucella*, *Patella*, *Conomurex*, *Valvata* and *Phorcus*) whereas others are more difficult to classify. The dashed lines separate Bivalvia and Gastropoda and it can be seen that very few Gastropoda examples are classified as Bivalvia (bottom left of the table). Although it appears at first sight that many more Bivalvia examples are classified as Gastropoda (top right), the actual numbers involved are small in most cases. For example, as there are only 4 examples for *Spondylus*, 25% corresponds to a single example. However, we did find that *Pecten*, *Spondylus*, *Cardium* and *Phorcus* had more within-class variance than other genera and the confusion between Bivalvia and *Patella* can be explained by a few *Patella* examples that could be considered outliers.

Within the Bivalvia examples, most confusion between genera is within the same order, i.e. Veneroida. Furthermore, all *Unio* samples that are not correctly classified are assigned to *Margaritifera* and vice versa. Both genera belong to the order *Unionoida*. There appears to be more confusion amongst the Gastropoda, although mainly within order Stylommatophora.

### 3.4 Reliability of classification

Supervised learning algorithms, i.e. algorithms that are trained to associate a particular output or class with particular input values, require data representing each possible output and any new sample will necessarily be associated with one of the classes used to train the algorithm. Kaufman and colleagues [41] described examples from classes other than those represented in the training set as “unclassifiable” and they investigated the sensitivity of the Coefficient of Similarity (CS) to indicate the reliability of their classification. Although the mean CS value was found to be higher for correctly classified shells than for incorrectly classified shells, there was significant overlap with some correctly classified shells having quite low CS values and some misclassifications having high CS values. Richter et al. [55] also considered measures of reliability in the classification of fish bone fragments. The probability of belonging to each class in the training set was calculated and used to provide a measure of confidence in the classification.

**Figure 4:**
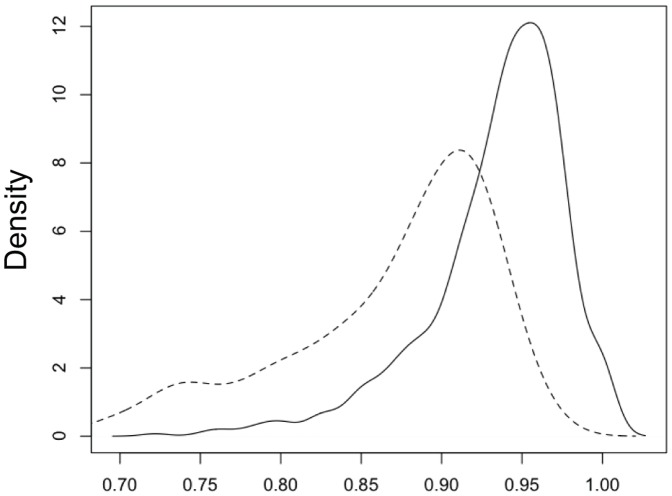
Coefficient of similarity distributions. The distributions of CS values for correct (solid line) and incorrect (dashed line) classifications.

Following Kaufman et al. [41], we considered the distribution of CS values for correct and incorrect classifications. We used a set of LVQ vectors obtained from all data in the 26 genera used in section 3.3 as the reference set in order to obtain CS values. Figure 4 shows frequency distributions (smoothed using a Gaussian kernel) for both correct and incorrect classifications. We found the greater number of genera in our study led to even more overlap between values than reported by Kaufman et al. [41]. A threshold of 0.91 on the CS value resulted in 133 of 622 (21%) correctly classified shells being rejected as unreliable and 53 of the 149 (36%) of the incorrect classifications being accepted. The LVQ vectors were also used to classify the examples of *Anisus*, *Rissoa* and *Antalis*. As these genera were not represented in the reference, they cannot be classified correctly. Table 2 shows how these examples were classified, together with the CS values. From the CS values, we might be inclined to accept the classification of one *Anisus* example as *Planobarius* and the classification of *Rissoa* as *Valvata* and *Conomurex*. In fact *Anisus* and *Planobarius* belong to the same family, Planorbidae, and *Rissoa* and *Conomurex* are both Littorinimorpha. The CS values for the other classifications are lower but still do not clearly identify the classifications as incorrect, given that correct classifications were found to have equally low values. Although the CS value may give some indication of reliability, it should be used with caution to assess classifications.

**Table 2:** Description of “unclassifiable” examples.

Real genus	Predicted genus	CS value
*Anisus*	*Valvata*	0.899
*Anisus*	*Planobarius*	0.944
*Rissoa*	*Valvata*	0.950
*Rissoa*	*Strombus*	0.944
*Antalis/Dentalium*	*Unio*	0.911
*Antalis/Dentalium*	*Unio*	0.887

The predicted genera for the “unclassifiable” examples and their Coefficient of Similarity (CS) values.

### 3.5 Classification of the Great Cornard beads

The same set of LVQ vectors were also used to classify six Great Cornard beads. As a whole artefact, bead 3682 could not be used for destructive analysis and therefore was not included in the amino acid analysis (see Supporting Information S2 and S3).

Five beads (4283 with two sub-samples, 3852, 4162, 3688 and 3884) were classified as *Nucella*. With CS values between 0.865 and 0.89, these classifications might be considered unreliable. However, 14% of all correct classifications also had CS values below 0.89 so we cannot rule out *Nucella* as the raw material for these beads. Furthermore, although the CS values are relatively low for the beads, the *Nucella* samples in our dataset are always classified correctly and that no other genera are classified incorrectly as *Nucella* (Figure 3). In other words, both the sensitivity and the specificity for *Nucella* appear very high. Although we do not claim that the five beads are definitely made from shells of the *Nucella* genus, we can say that amongst all the genera in our training set, *Nucella* is the most likely, with CS values to the second best match (various taxa) ranging from 0.798 to 0.851, with mean difference −0.042 from *Nucella*. The beads could of course be made from shells of some genus not currently represented in our database, but we suggest that this genus would be closely related to *Nucella*.

Although certainly not the only taxon exploited for ornamental or technological purposes, shell ornaments are very often identified as *Spondylus* [19], [56]–[59]. *Spondylus* therefore could have been a potential candidate for the Great Cornard beads. *Spondylus* are not well-represented in the training set, but principal component analysis shows that the amino acid composition of *Spondylus* does not overlap with that of the beads. Furthermore, *Nucella* shells are never confused with *Spondylus*. Thus it seems very unlikely therefore that the shell beads could actually be *Spondylus*.

The sixth bead (3870) was classified as *Unio* with a CS value of 0.89. This bead could be classified as *Antalis*, which was not included in the training set; nonetheless, CS values between bead 3870 and the two *Antalis* examples were 0.9 and 0.91, showing that this genera is a closer match than *Unio*.

### 3.6. Morphology

**Figure 5:**
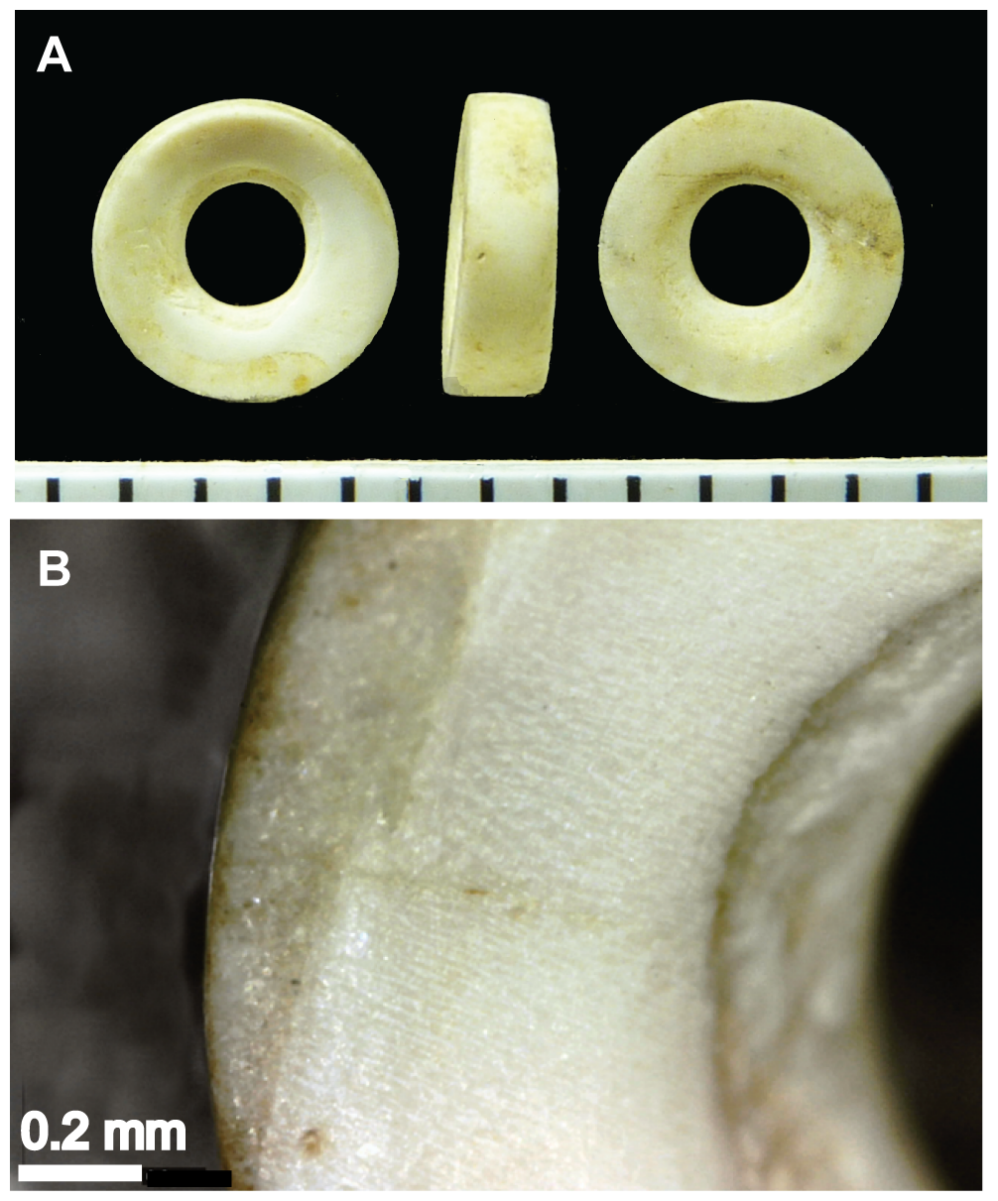
Great Cornard bead 3682. (a) Photograph and (b) photomicrograph of the surface. The bulk of the bead is granular but this surface has the remnants of a thin layer of a whiter, more opaque material with a finely striated appearance.

**Figure 6:**
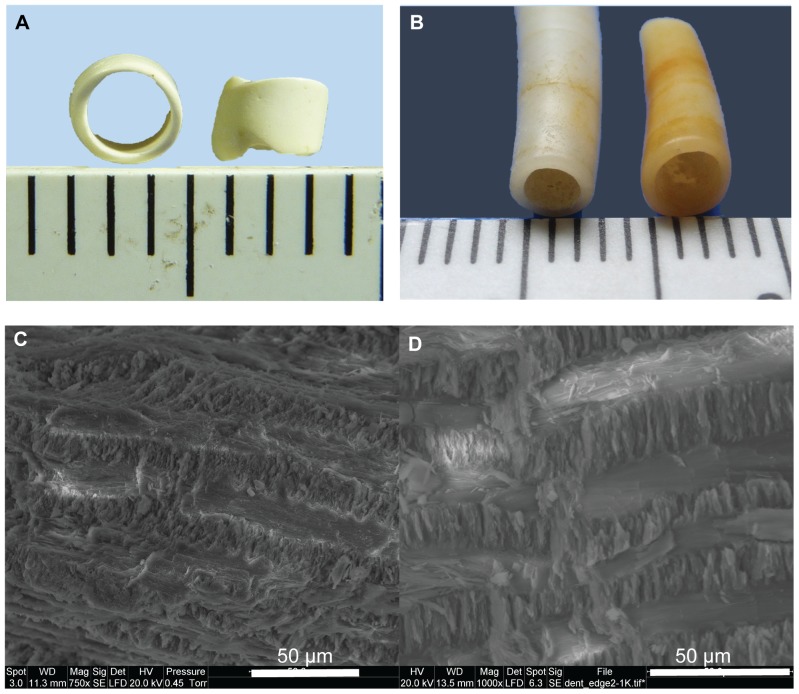
Great Cornard bead 3870 and *Antalis* sp. Photograph (a, b) and scanning electron microscopy images (c, d) of bead 3870 (a, c) and *Antalis* sp. (b, d).

Optical and SEM analyses were undertaken to investigate the macro- and micro-structure of the Great Cornard beads. The beads were between 4 and 5.5 mm in diameter, with a central perforation of ∼2 mm and maximum thickness of ∼2 mm. Five of the beads sampled in the amino acid study were similar in shape to bead 3682 and each had the remnants of a thin layer of a whiter, more opaque material on one face (Figure 5). This layer had a finely striated appearance and cross-laminar architecture, whereas the bulk of these beads comprised a fine granular, apparently homogenous and relatively translucent material. Bead 3870 showed no evidence of this layer and differed in shape from the other five beads (Figure 6). The appearance of this bead suggested it might be a section cut from a tubular shell without further working. A description of the macroscopic and microscopic features observed in each of the six Great Cornard beads is given in the Supporting Information S3.

The microstructures of the beads, observed by SEM, were compared with those of three candidate shells; *Spondylus gaederopus*, *Nucella lapillus* and *Antalis* sp. (Supporting Information S3). *Spondylus* was selected for analysis due to its extensive use in jewellery and ornament creation throughout prehistory, whilst the results of the amino acid analysis suggested *Nucella* as a potential candidate. Its availability on shores in the UK made *Antalis* a possibility for bead 3870.

Although it is not possible to identify the mollusc species concerned based on the SEM analysis, we can conclude that, whilst *Spondylus* cannot entirely be ruled out on this evidence, *Nucella* seems to be a closer match for the features observed in five of the beads: a granular, homogeneous, calcitic structure with a thin layer of cross-lamellar structure to one edge. The analysis also shows that the microstructure of *Antalis* is very similar to that of bead 3870, which appeared to be entirely cross-lamellar (Figure 6).

### 3.7 Raman spectroscopy

Raman spectroscopy was applied to 15 modern and 2 fossil *Nucella* sp. specimens, a modern *Antalis* sp. shell and the six beads samples. Spectra were obtained for both the interior and exterior surfaces of each shell. Many molluscan taxa lay down alternate microstructural layers of the bio-polymorphs of calcium carbonate (calcite and aragonite), whilst others may display one phase only [60]. Although *Nucella* shells have been reported as calcite only [61], Raman spectroscopy identified aragonite in the tip, lip and innermost layer, with calcite identified in the external and middle regions of the shell (Supporting Information S4). For the *Antalis* sp. shell, aragonite was found as the only polymorph present in both the interior and exterior regions.

Calcite only was observed in beads 3688 and 3852, whilst calcite and aragonite were both identified in samples 3884, 4162, and 4283. The exception was the bead fragment 3870, which was identified as aragonite only (Supporting Information S4).

## Discussion

Although differences in amino acid compositions between molluscan genera have been shown by others and exploited as a taxonomic identification tool [38], [40]–[41], we should not necessarily expect the bulk quantitative values of the amino acid signature to preserve the same level of taxonomic information as protein sequences. Confounding factors, such as age, temperature and environment undoubtedly increase the variance within genera, but our analysis has shown no consistent patterns that can be modelled for the taxa selected (section 3.2). Moreover, our dataset includes a number of different species for some genera and examples of unknown species for others. Nonetheless, bulk amino acid compositional data from the intra-crystalline fraction of proteins within mollusc shells preserves taxonomic information. Our analysis has shown that the amino acid signatures of Bivalvia and Gastropoda are generally distinct. Furthermore, most misclassifications occur due to confusion between genera of the same order. Whereas just over 77% of examples are classified correctly at genus level, over 84% are correct at the level of order. Whilst ∼11% of Bivalvia are incorrectly classified as Gastropoda, less than 2% of Gastropoda are classified as Bivalvia. The effect of different class sizes cannot be ignored. Although the NEaar database provides valuable taxonomic information, the low number of samples available for many taxa adversely affects the classification; future studies should include more samples and the database extended to improve the level of confidence. Nevertheless we have demonstrated that differentiation is possible and that closely related genera have similar amino acid signatures.

**Figure 7:**
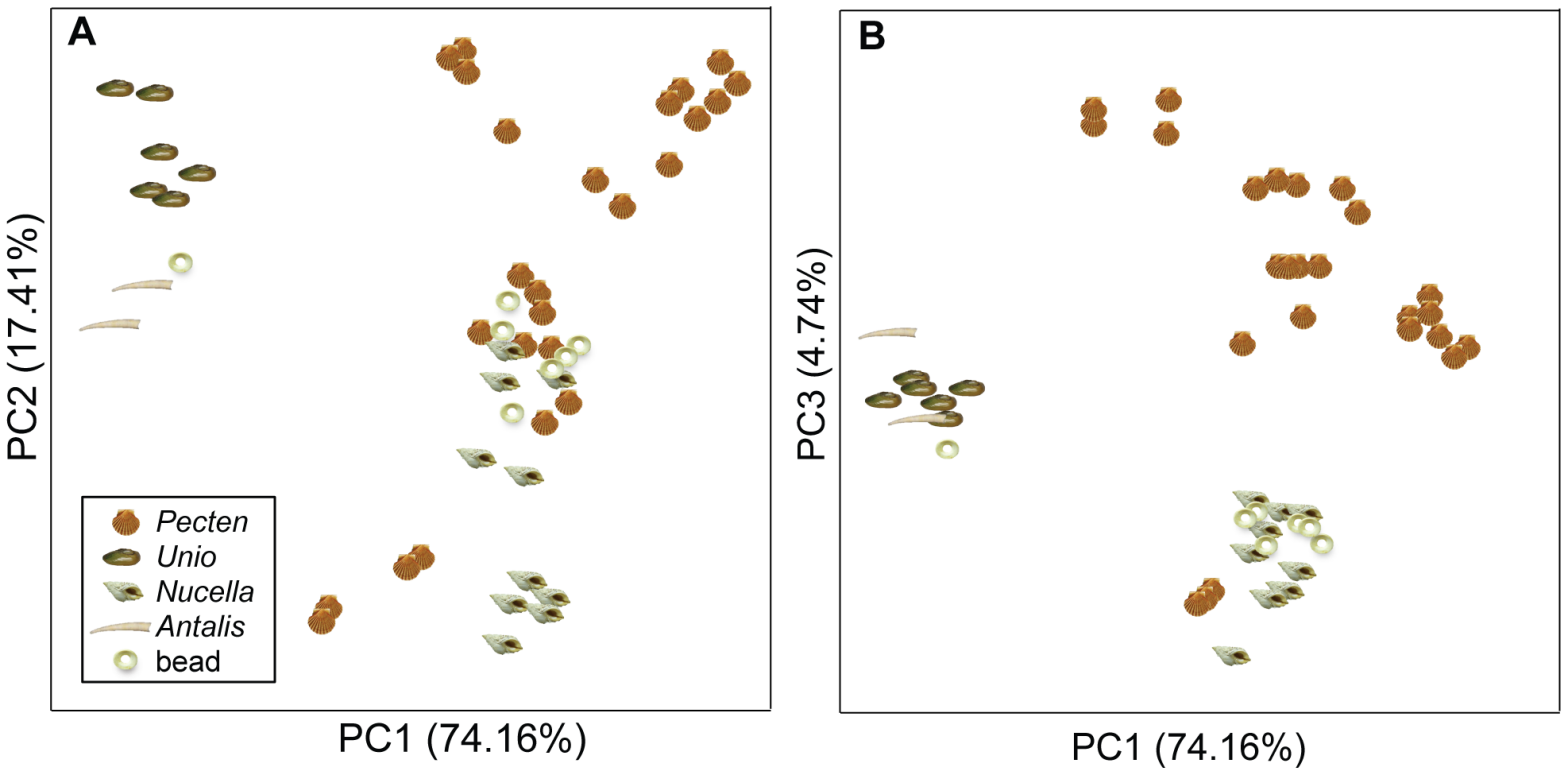
PCA scores plots showing examples closest to the beads. Scores plots from principal components analysis showing only examples from genera with scores closest to the beads. Bead 3870 can be seen, clustered with *Unio* and *Antalis* examples on the left of the plots. Although *Pecten* examples overlap with *Nucella* and the other beads in the scores plot for the first two principal components (a), separation can be seen along the third component (b).

As an application, we investigated the possible molluscan taxa (among those represented in our dataset) that might have been used as the raw material for the shell beads found at the site of Great Cornard. Principal components analysis revealed clusters in the amino acid data and the scores plots in Figure 7 include only the genera with the highest levels of similarity to the Great Cornard beads. The plots show:

- The similarity of bead 3870 to *Antalis* and *Unio*;
- The similarity of the remaining bead samples to both *Pecten* and *Nucella* for the first two principal components;
- The separation between *Pecten* and the beads on the third component and the variation within the *Pecten* samples. This genus has particularly high within-groups variance.

**Figure 8:**
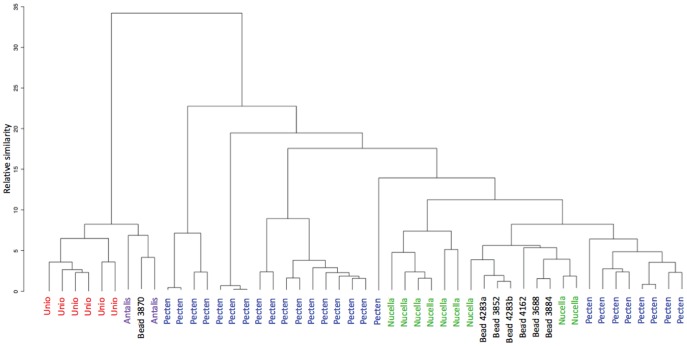
Dendogram for the genera most similar to the beads. Dendogram showing the results of hierarchical cluster analysis on the amino acid signatures for the genera most similar to the Great Cornard beads.

Hierarchical cluster analysis on these same examples shows the different clusters of *Pecten*, whilst bead 3870 clusters with *Antalis* and *Unio* and the rest of the beads with *Nucella* (Figure 8).

For any classification problem where all classes cannot be represented, classification will only ever be able to provide the most likely class of those used in training. Thus, although the Great Cornard beads may belong to a taxon not included in our database, we can confidently say that this hypothetical taxon is likely to be closely related to *Nucella*, and that many of the genera for which we have amino acid data, including *Spondylus*, can be excluded from the range of possibilities.

Five of the beads (including bead 3862 shown in Figure 5) were very similar in shape and low power microscopy shows that they all have the remains of a layer of whiter material on one surface of the disc. SEM reveals the bulk of each bead to be a more or less homogenous material and the whiter layer to be have a cross lamellar structure. Raman spectroscopy provides evidence for both calcite and aragonite in four of these beads, as well as identifies both of these minerals in the *Nucella* shells. Therefore, *Nucella* or a similar genus, cannot be ruled out on this evidence. On the other hand, Raman spectroscopy identifies only aragonite in both the *Antalis* shell and bead 3870. Together with the similarity in microstructure revealed by SEM and in amino acid composition, this strongly supports the idea that this bead may be a section cut from a tusk shell.

## Conclusions

We have investigated the potential of a biomolecular approach based on the analysis of the amino acid profiles of intra-crystalline molluscan proteins for taxonomic identification. This is a fast and cost-effective method with minimal sample requirement (<2 mg powdered shell) and would therefore be a viable analytical tool for the investigation of precious artefacts with minimal destruction. As we always assess chiral amino acid distribution in these analyses, the same data set may have geochronological value.

A dataset of 777 samples (Supporting Information S5) was used, representing 29 genera from 27 families and 15 orders. Samples are Bivalvia and Gastropoda with the exception of the two *Antalis* samples, which are Scaphopoda. Although this does not represent all possible taxa, and therefore our method cannot give a definitive identification, we show that taxonomic information is preserved in the bulk amino acid composition. Although we did not attempt to model the effect of age or temperature, we have shown that the stable IcP fraction can be used as a chemotaxonomic tool. Therefore, if a sample of unknown taxonomic origin is analysed, it can be matched to the most closely related taxa (from those available), whilst other taxa can be discounted.

Proteomic analysis has been applied to mollusc shells [62]–[63] and, although currently requiring larger samples sizes, could potentially provide more definite taxonomic identification. However, for a PMF (peptide mass fingerprinting) approach, classification would require a database with sufficient examples from any class we would hope to recognise. For tandem mass spectrometry (MS/MS) analyses, an important requirement is that protein sequence data are available for a wide range of taxa, which is currently not the case. However, in the future we hope to use mass spectrometry to confirm or rule out putative identifications from the amino acid method.

We applied our analyses to six beads from an Early Bronze Age burial at Great Cornard, Suffolk (UK). The integration of biomolecular analyses with morphological observations and mineralogical investigations has allowed us to shed light on the natural resources exploited by the people who made the Great Cornard shell ornaments in the past. We have been able to:

- exclude *Spondylus* as the raw material used to create the beads;
- demonstrate that at least two different taxa were selected;
- suggest *Antalis* as the raw material for bead 3870, on the basis of the amino acid analyses, morphological characterisation and Raman spectroscopy;
- show that the other five beads are similar to each other in macroscopic appearance, mineralogy and amino acid profiles;
- hypothesise that the raw material used for these five beads might have been one or more species with amino acid fingerprints similar to *Nucella* or a closely-related taxon.

Currently *Nucella* and *Antalis* are found along UK shores, with *Nucella* abundant around the Suffolk coast and *Antalis* less widespread but present along the Southern coast [64]. The use of both tusk shells (*Dentalium*/*Antalis*) and dogwhelk (*Nucella*) as personal ornaments has been documented in archaeological sites since the Upper Palaeolithic [15], [65]–[66] and their presence at Great Cornard as raw material for worked beads may therefore be of particular cultural significance.

## Supporting Information

Supporting Information S1 Further investigation of the effect of age and geographical region on amino acid composition.
(PDF)

Supporting Information S2 Amino acid analyses of the Great Cornard beads.
(PDF)

Supporting Information S3 Optical microscopy and SEM analyses.
(PDF)

Supporting Information S4 Raman spectroscopy.
(PDF)

Supporting information S5 Amino acid dataset.
(XLSX)
