# Supplementary material for: An Integrated Approach to the Taxonomic Identification of Prehistoric Shell Ornaments
Source: PLoS One. 2014 Jun 17;9(6):e99839. doi: 10.1371/journal.pone.0099839 (PMC4061022; doi:10.1371/journal.pone.0099839)
Supplement: Supporting Information S1 — Further investigation of the effect of age and geographical region on amino acid composition. (PDF) [file pone.0099839.s001.pdf]

SI-1: Further investigation of the effect of age and geographical region on amino acid composition.

Figure S1 shows the relative amino acid compositions for 78 samples of the species *Valvata piscinalis* with ages ranging from 500 to 600,000 years (approximate dates in years are derived from the estimate age of the marine isotope stages, MIS, see SI-5). All shells were obtained in the UK and show no consistent trend with age.

In addition to *Patella* and *Valvata*, data were available for different ages for three further genera: *Arctica* (although all ages are < 200 years), *Littorina* (ranging from 500 to ca. 2,000,000 years) and *Margaritifera* (also < 200 years). Figure S2 shows the concentrations for aspartic acid/asparagine (Asx) and serine (Ser) for these genera. Although the plots seems to show increased variation in relative concentration for the older *Littorina* shells, particularly for serine, this is similar to the level of variance in *Margaritifera* samples, which are much younger. The variation in *Littorina* samples could be explained by the fact that several different species are included: *L. littorea*, *L. obtusata* and *L. saxatilis* as well as undetermined species. However, this does not explain the variation in *Margaritifera* samples, which are all of the species *M. margaritifera*. There is no consistent trend with age in any of the three genera for either Asx or Ser. For these genera, the other four amino acid concentrations show even less relationship with age than for *Patella*.

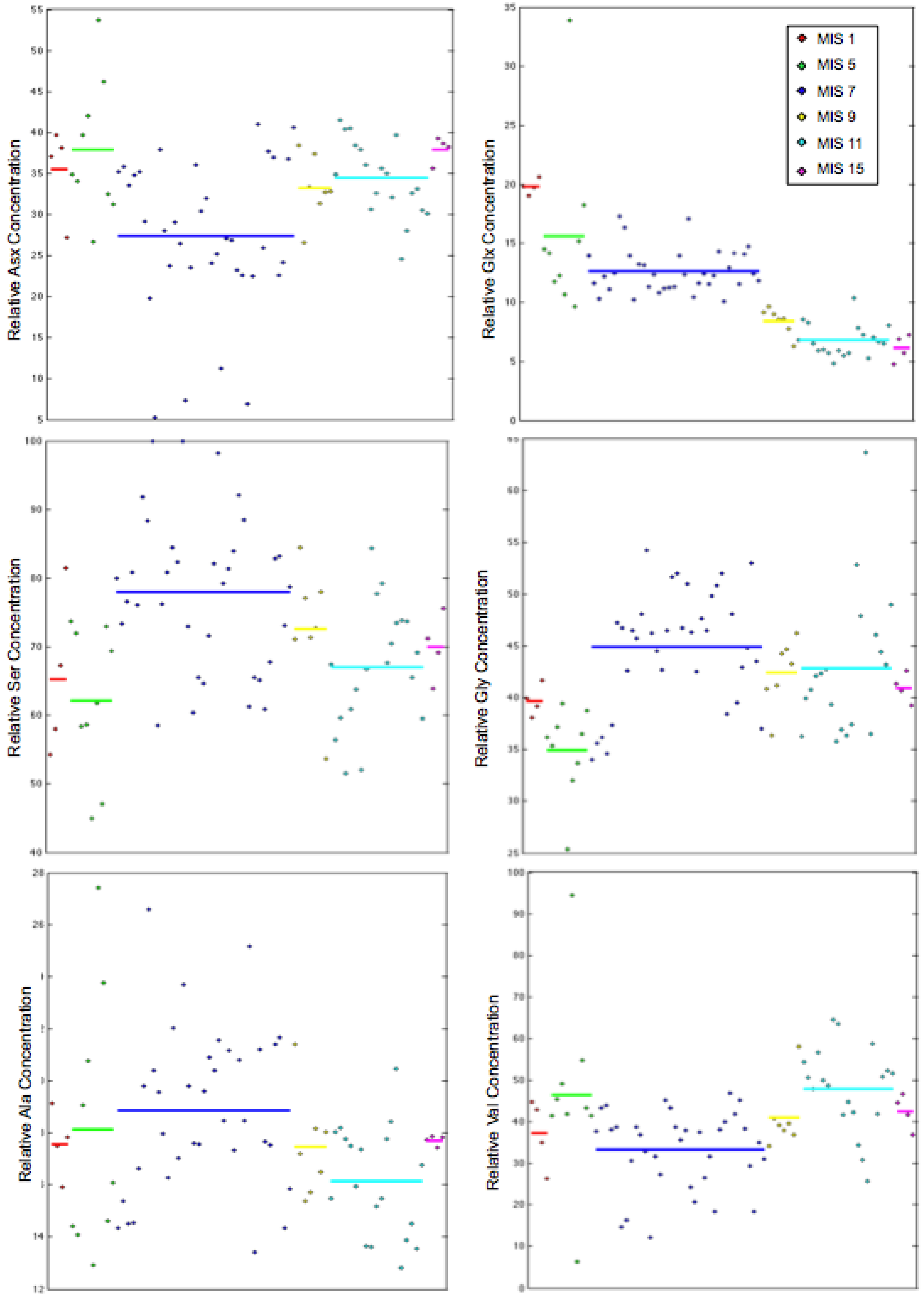

Figure S-1: The relative concentrations of the six amino acids used in the analysis for 78 *Valvata* examples of different ages.

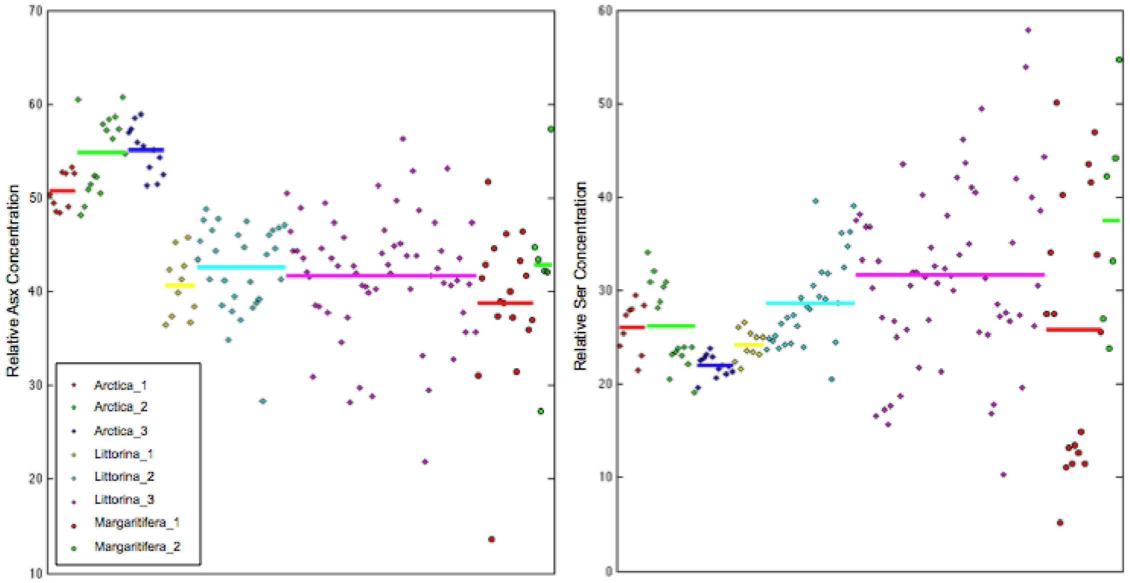

Figure S-2: The relative concentrations for Asx (aspartic acid/asparagine) and Ser (serine) plotted by age for samples in the genera *Arctica*, *Littorina* and *Margaritifera*. The samples are grouped by age as Arctica_1 (0-50 years), Arctica_2 (50-100 years), Arctica_3 (100-200 years); Littorina_1 (ca. 500 years), Littorina_2 (ca. 125,000 years, MIS 5), Littorina_3 (ca. 250,000 years, MIS 7); Margaritifera_1 (0-100 years), Margaritifera_2 (100-200 years).
