# Supplementary material for: An Integrated Approach to the Taxonomic Identification of Prehistoric Shell Ornaments
Source: PLoS One. 2014 Jun 17;9(6):e99839. doi: 10.1371/journal.pone.0099839 (PMC4061022; doi:10.1371/journal.pone.0099839)
Supplement: Supporting Information S2 — Amino acid analyses of the Great Cornard beads. (PDF) [file pone.0099839.s002.pdf]

SI-2 Amino acid analysis

Table S-1: Normalised amino acid compositions for the Great Cornard beads.

Bead ID	Asx	Glx	Ser	Gly	Ala	Val
4283	42.57 ±0.02	9.32 ±0.38	6.2 ±0.08	12.94 ±0.65	19.26 ±0.55	9.72 ±0.42
4283	43.61 ±0.14	9.84 ±0.06	5.72 ±0.13	12.76 ±0.3	18.55 ±0.0	9.52 ±0.24
3852	44.47 ±0.11	9.65 ±0.04	5.87 ±0.02	12.08 ±0.45	18.16 ±0.08	9.75 ±0.33
4162	39.32 ±0.58	8.97 ±0.02	6.4 ± 0.2	13.75 ±0.68	20.51 ±0.08	11.04 ±0.24
3688	40.23 ±0.31	10.55 ±0.18	6.45 ±0.27	16.56 ±0.13	17.17 ±0.67	9.04 ±0.21
3884	39.38 ±0.46	11.2 ± 0.08	6.26 ±0.07	16.05 ±0.68	18.15 ±0.13	8.96 ±0.06
3870	17.67 ±0.1	16.68 ±0.24	8.38 ±0.57	32.96 ±1.3	17.78 ±0.51	6.68 ±0.09

The total hydrolysable amino acid (THAA) normalised concentrations are reported as the mean and standard deviation of the two analytical replicates obtained for each sample. Two sub-samples were analysed from bead 4283.
