# Supplementary material for: An Integrated Approach to the Taxonomic Identification of Prehistoric Shell Ornaments
Source: PLoS One. 2014 Jun 17;9(6):e99839. doi: 10.1371/journal.pone.0099839 (PMC4061022; doi:10.1371/journal.pone.0099839)
Supplement: Supporting Information S3 — Optical microscopy and SEM analyses. (PDF) [file pone.0099839.s003.pdf]

SI-3: Optical microscopy and SEM analysis

Comparative Specimens

Spondylus gaederopus **Linnaeus, 1758*:*** the shell is formed from both calcite and aragonite. The spines and outer part of the shell are formed from calcite, whilst the inner part and the hinge area are formed from layers of aragonite. The calcite is laid down successively at the shell margin by the very edge of the mantle, whilst the aragonite is formed in columns across the whole of the inner surface of the shell at its interface with the mantle. The aragonite is cross lamellar, the columns of needles alternating in direction in adjacent columns so that they meet at an angle of 90-110 degrees when viewed parallel to the shell margin. When viewed along the main growth axis this produces a striped effect termed the zebra-fur optic [1].

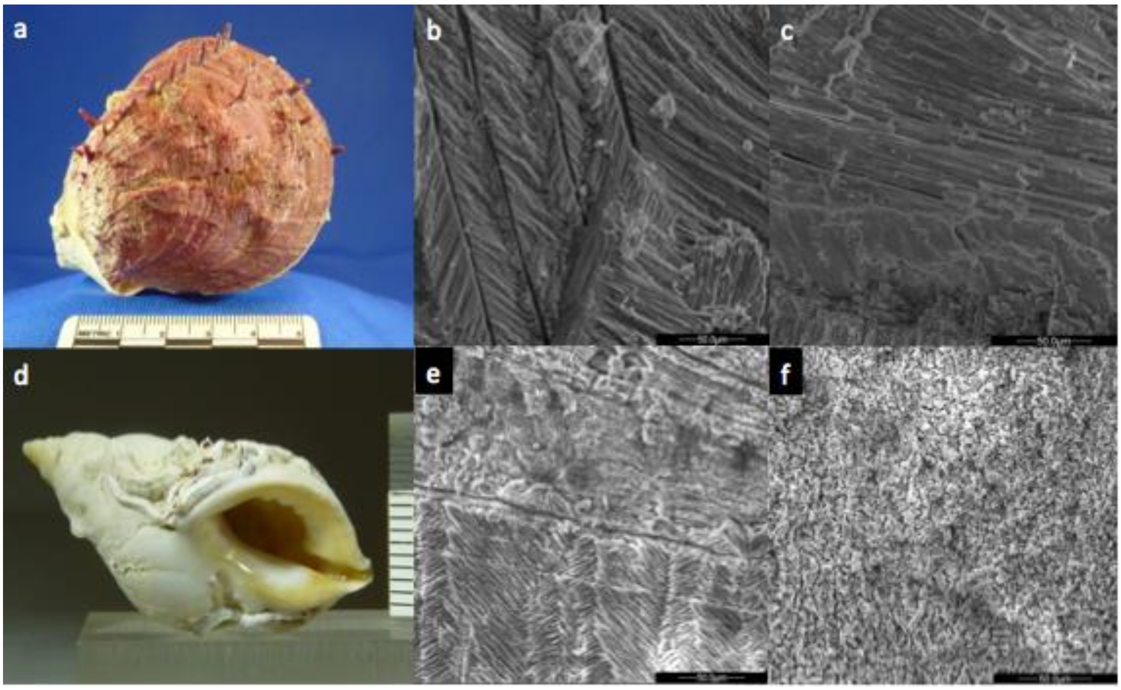

Figure S-3: The microstructures of two possible candidate shells used as raw material for the Great Cornard beads: (a) *Spondylus* and (d) *Nucella*. (b) Cross-lamellar structure of *Spondylus.* (c) Overlying structure perpendicular to the shell edge (fracture surface). (e) Cross lamellar structure of *Nucella* and (f) overlying structure perpendicular to the shell edge (etched sections).

***Nucella lapillus* Linnaeus, 1758** has shells consisting of two microstructural types; a thick outer homogeneous layer of calcite and a thin inner layer of crossed lamellar material. The crossed lamellar shell is stronger than the homogenous shell but is energetically more expensive to produce, and *Nucella* on shores with high predation risk are protected by a disproportionately thickened homogeneous layer, providing more than sufficient material for bead production [2]. The needles in the cross-lamellar layer in *Nucella* appear to meet at a more oblique angle than in *Spondylus*.

The Great Cornard beads

The small cross-sectional areas do not provide a comprehensive view of the features or the variability in structure of the shell beads. This is compounded by the diagenetic deterioration and the loss, due to the working of the material, of surface morphology that might have provided evidence of the orientation of the observed features relative to the exterior and interior surfaces of the shell.

Bead	Description	Microstructure	SEM
3682	Mostly a granular, relatively translucent material showing curved incremental growth lines on one side (Fig. S-1) whilst the opposite is covered in a thin layer of a whiter, more opaque material with a co-axially organised crystalline appearance (Fig. 5).	Bead intact	Bead intact
3688	Similar 3682 in size, shape and layers but the whiter layer is not continuous over the worked surface.	The bulk seems to show a hint of columns of differently oriented prisms perpendicular to the growth lines. The other layer did not feature in the sections viewed by SEM.	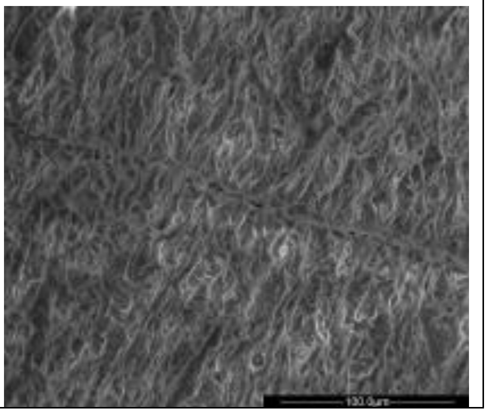

3852	Similar in size and shape to 3682 but a relatively opaque chalky white appearance and quite friable with no visible layering.	Appears as a homogenous decayed calcite structure. Possibly a hint of columns of differently aligned calcite crystals.	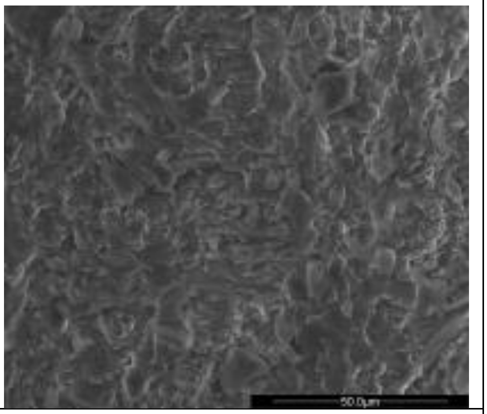
3870	Very different in shape, half the diameter of 3682 and very thin walled. Opaque white material with no visible layering (Fig. 6).	Entirely cross-lamellar structure. Columns of prisms aligned parallel to the diameter of the bead.	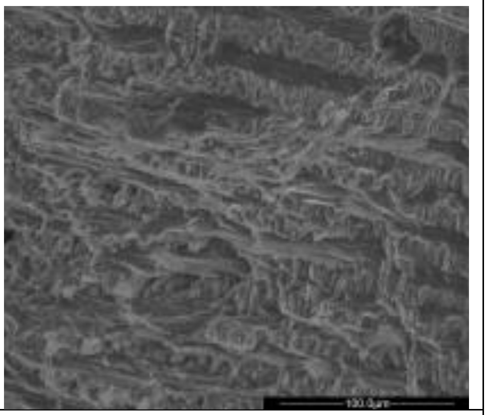
3884	Similar to 3682 in size, shape and layers.	The bulk is homogenous and the thinner layer is cross-lamellar.	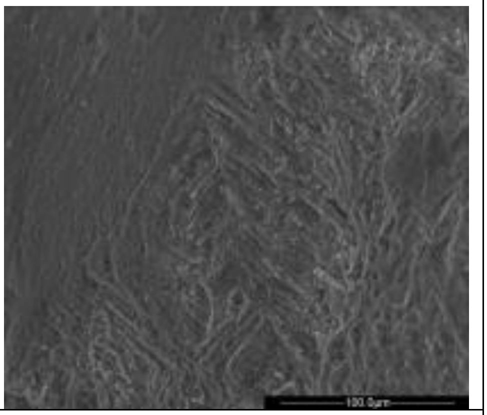
4162	Thin dished bead with equal thicknesses of a granular (convex surface and a more opaque white material (concave surface).	Difficult to see separate layers but appears to be rough columns of differently aligned calcite crystals.	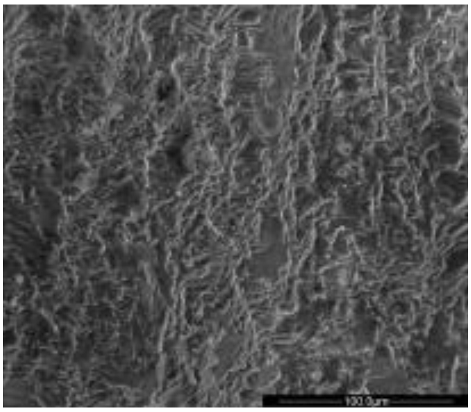

4283	Similar to 3682 in size, shape and layers but granular layer not so well preserved.	Layers of differently aligned calcite crystals.	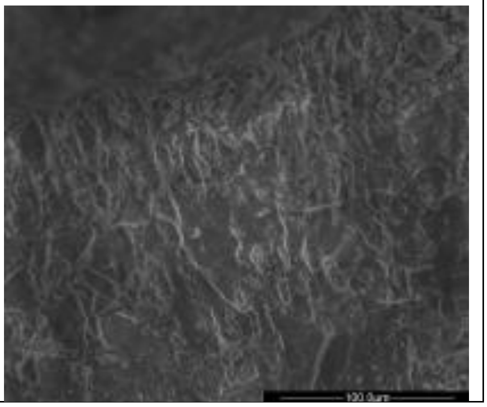

Table S-3: Description of the macroscopic and microscopic features observed in each of the seven Great Cornard beads.

References

1. Maier E and Titschack J (2010) *Spondylus gaederopus*: A new Mediterranean climate archive—Based on high-resolution oxygen and carbon isotope analyses. Palaeogeography, Palaeoclimatology, Palaeoecology 291: 228-238.
2. Avery R and Etter RJ (2006) Microstructural differences in the reinforcement of a gastropod shell against predation. Marine Ecology Progress Series 323: 159-170.
