# Supplementary material for: An Integrated Approach to the Taxonomic Identification of Prehistoric Shell Ornaments
Source: PLoS One. 2014 Jun 17;9(6):e99839. doi: 10.1371/journal.pone.0099839 (PMC4061022; doi:10.1371/journal.pone.0099839)
Supplement: Supporting Information S4 — Raman spectroscopy. (PDF) [file pone.0099839.s004.pdf]

SI-4 Raman spectroscopy

Raman spectroscopy was used to investigate the following samples; fifteen modern *Nucella* sp. shells from three different sites (Skye, Devon and Craster with five shells per site), one modern *Antalis* sp. shell, two fossil *Nucella* sp. shells (S1 and S2) from Easington Raised Beach, and six archaeological bead fragments ~2 mm in size (3688, 3852, 3884, 3870, 4162, and 4283).

Raman spectra were collected from the external regions of the shells with single measurements in sections M1, M2, M3, M4 and M5 (Figure S-4a) (in total five spectra per shell). Raman spectra were also collected from the middle and internal regions of the modern and fossil *Nucella* sp. shells sectioned lengthwise (Figure S-4b) (six spectra), and from the inside of the modern *Antalis* sp. shell (three spectra). Three spectra were collected from each side of the archaeological bead fragments.

Bio-polymorphs of calcium carbonate (aragonite and calcite) were identified in the Raman spectra by comparing the peak assignments obtained in this study with literature references [1-3].

Figure S-5 shows typical spectra of calcite found in the external region (position M3) of modern and fossil *Nucella* sp. shells. Aragonite was also found in the tip and lip regions of these shells (spectra not shown). In the middle layer of the modern and fossil *Nucella* sp. shells, calcite was identified, and in the internal layer, aragonite was identified (Figures S-6 & S-7). These results are typical of all of the *Nucella* sp. shells sampled in this study. For the modern *Antalis* sp. shell, only aragonite was found in both the external and internal parts of the shell (Figure S-8). For the archaeological bead fragments, only calcite was observed in samples 3688 and 3852, whilst calcite and aragonite were both observed in the 3884, 4162, and 4283 bead fragments (Figure S-9). The exception was bead fragment 3870, which was identified as aragonite only (Figure S-10).

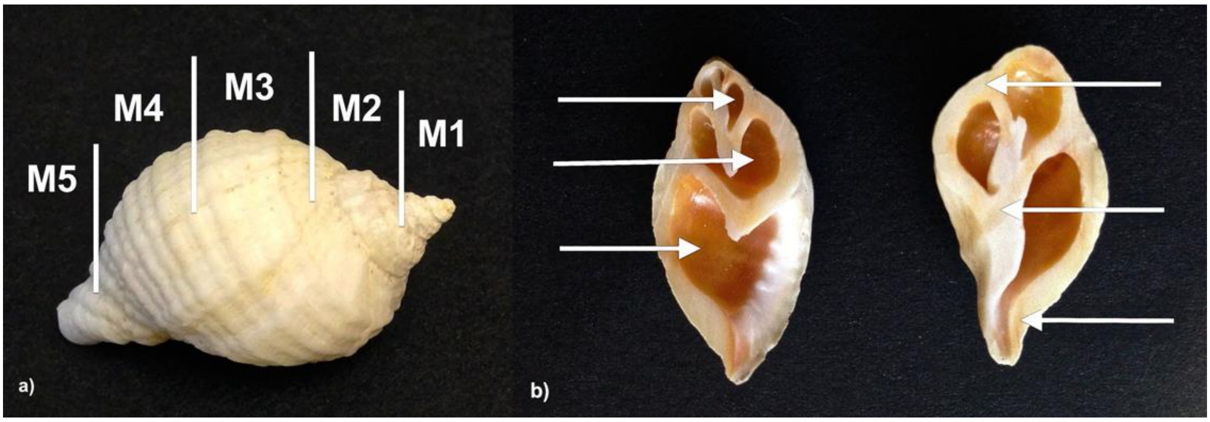

Figure S-4: (a) Raman spectra were obtained in the external part of the shells from regions M1-M5 (modern *Nucella* sp. shown as an example). (b) Picture of a sectioned modern *Nucella* sp. shell showing the areas where spectra where collected from the internal layer (left) and middle layer (right).

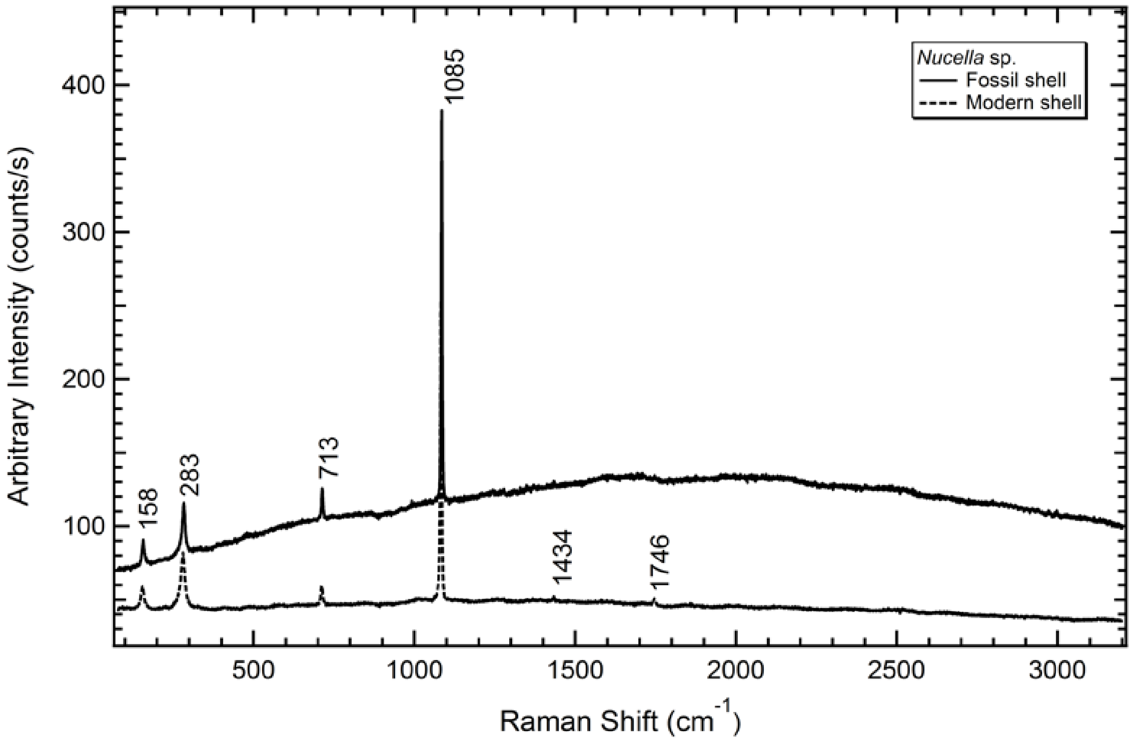

Figure S-5: Calcite spectra collected from the external region (M3) of a modern *Nucella* sp. shell from Devon and a fossil *Nucella* sp. shell (S2).

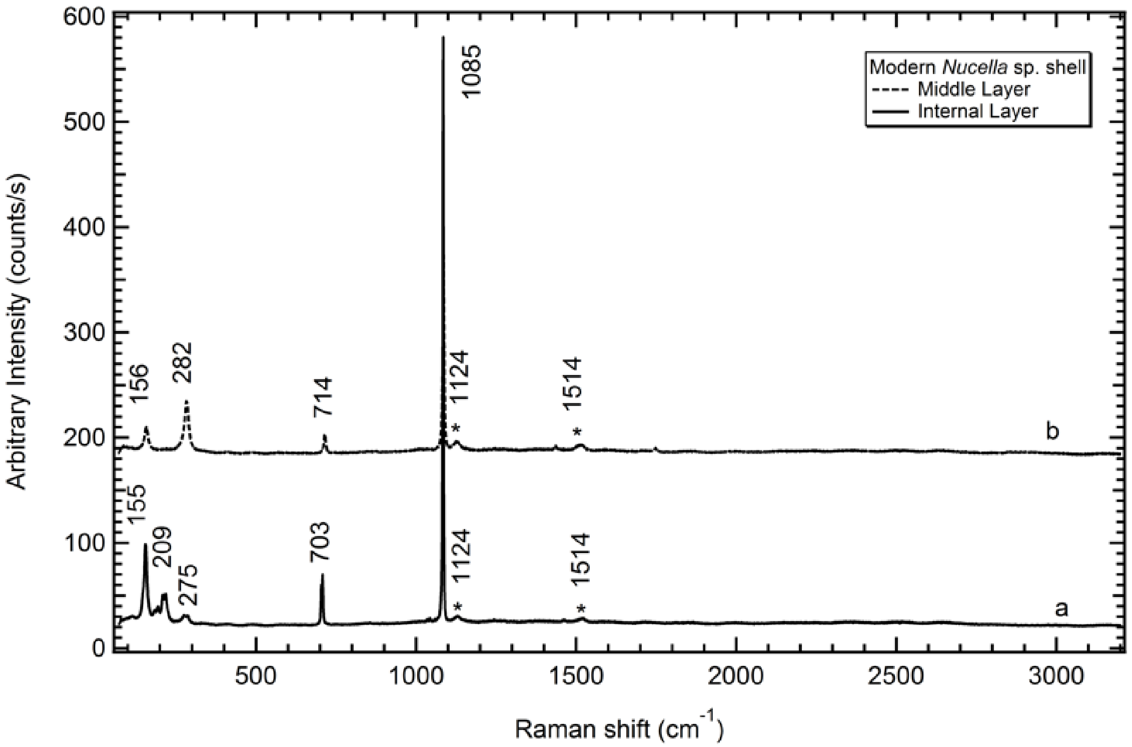

Figure S-6: (a) Aragonite spectrum obtained from the internal layer and (b) calcite spectrum obtained from the middle layer of a modern *Nucella* sp. shell from Devon. The positive identifications of aragonite and calcite were made by comparison with the literature [1-3]. Peaks marked with * correspond to carotenoids in the shell [4-5].

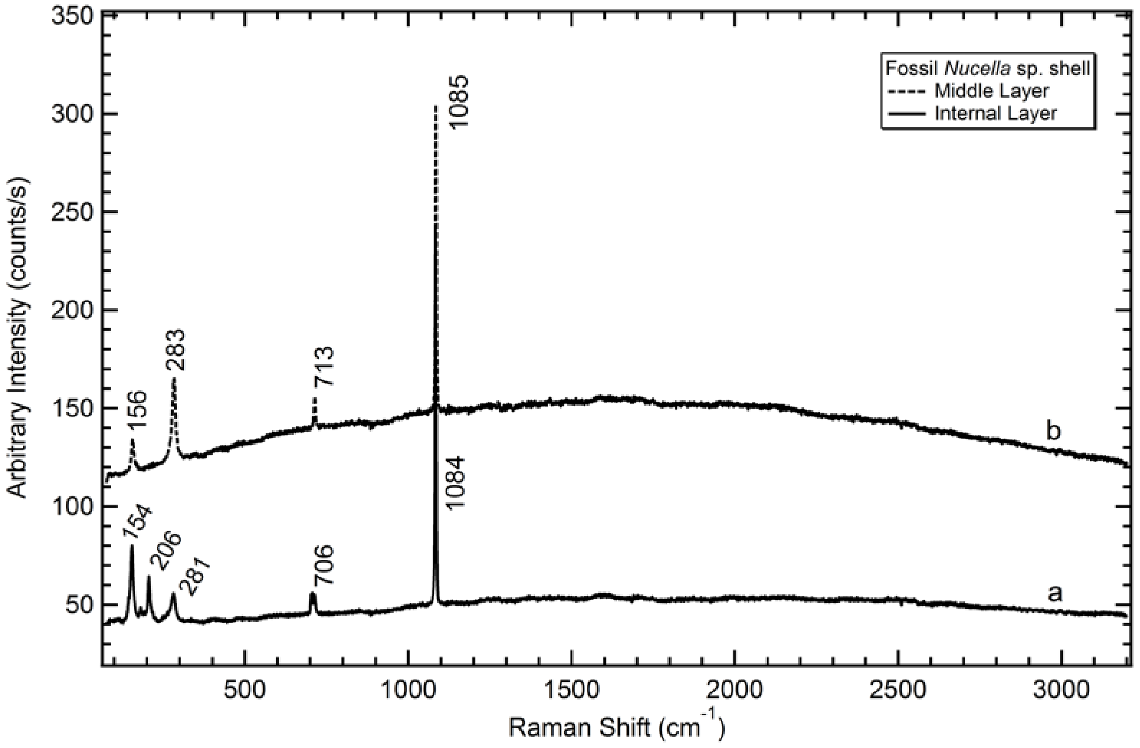

Figure S-7: (a) Aragonite spectrum obtained from the internal layer and (b) calcite spectrum obtained from the middle layer of the fossil *Nucella* sp. shell (S2). The positive identifications of aragonite and calcite were made by comparison with the literature [1-3].

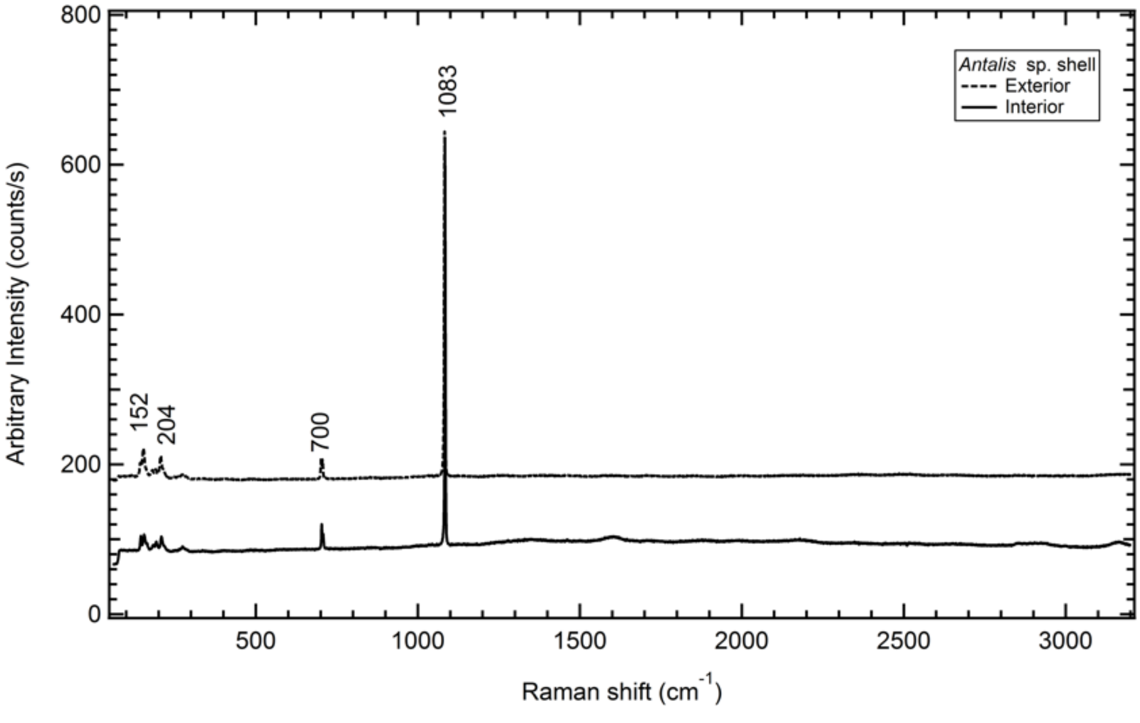

Figure S-8: Aragonite spectra found in the exterior (M3) and interior regions of a modern *Antalis* sp. shell. The positive identification of aragonite was made by comparison with the literature [1-3].

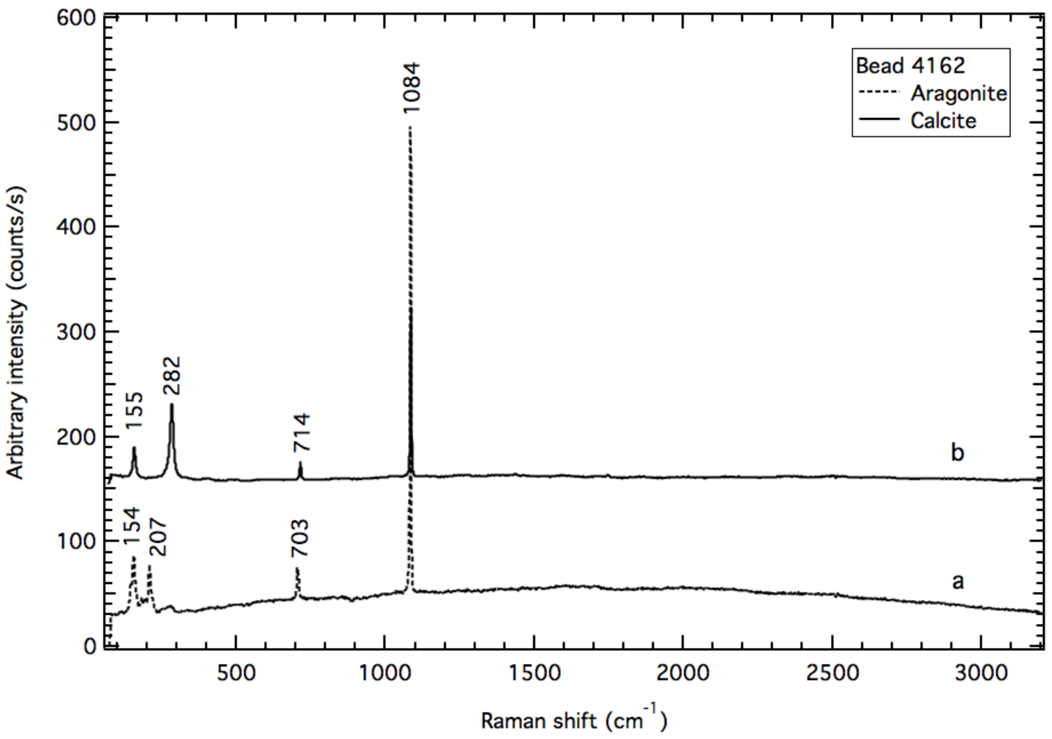

Figure S-9: (a) Aragonite and (b) calcite spectra found on opposite sides of the 4162 archaeological bead fragment of unknown shell origin. The positive identifications of aragonite and calcite were made by comparison with the literature [1-3].

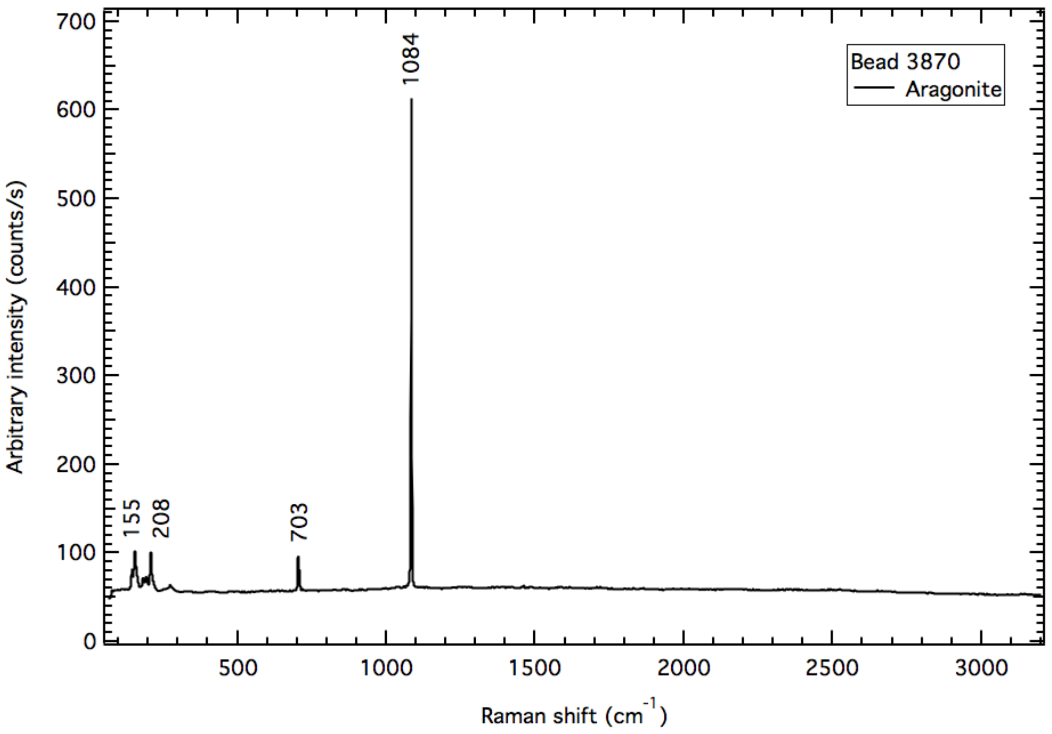

Figure S-10: Aragonite spectrum obtained from the 3870 archaeological bead fragment of unknown shell origin. The positive identification of aragonite was made by comparison with the literature [1-3].

References

1. Clode P, Lema K, Saunders M and Weiner S (2011) Skeletal mineralogy of newly settling *Acropora millepora* (Scleractinia) coral recruits. Coral reefs 30: 1-8.
2. Urmos J, Sharma SK, Mackenzie FT (1991) Characterization of some biogenic carbonates with Raman spectroscopy. American Mineralogist 76: 641-646.
3. Parker J, Thompson S, Lennie A, Potter J and Tang C (2010) A study of the aragonite-calcite transformation using Raman spectroscopy, synchrotron powder diffraction and scanning electron microscopy. CrystEngComm 12: 1590-1599.
4. Hedegaard C, Bardeau JF, and Chateigner D (2006). Molluscan shell pigments: an in situ resonance Raman study. Journal of Molluscan Studies 72:157–162.
5. Withnall R, Chowndhry BZ, Silver J, Edwards HGM and de Oliveira LFC (2003). Raman spectra of carotenoids in natural products. Spectrochimica Acta Part A, 59:2207–2212.
